# Insulation Cork Boards—Environmental Life Cycle Assessment of an Organic Construction Material

**DOI:** 10.3390/ma9050394

**Published:** 2016-05-20

**Authors:** José D. Silvestre, Nuno Pargana, Jorge de Brito, Manuel D. Pinheiro, Vera Durão

**Affiliations:** 1 CERIS, Instituto Superior Técnico, Universidade de Lisboa, Av. Rovisco Pais 1, Lisboa 1049-001, Portugal; jb@civil.ist.utl.pt (J.d.B.); manuel.pinheiro@tecnico.ulisboa.pt (M.D.P.); vera.durao@tecnico.ulisboa.pt (V.D.); 2 Instituto Superior Técnico, Universidade de Lisboa, Av. Rovisco Pais 1, Lisboa 1049-001, Portugal; nuno.pargana@ist.utl.pt

**Keywords:** biogenic carbon, cradle to cradle, environmental impact, insulation cork boards, life cycle assessment, thermal insulation materials

## Abstract

Envelope insulation is a relevant technical solution to cut energy consumption and reduce environmental impacts in buildings. Insulation Cork Boards (ICB) are a natural thermal insulation material whose production promotes the recycling of agricultural waste. The aim of this paper is to determine and evaluate the environmental impacts of the production, use, and end-of-life processing of ICB. A “cradle-to-cradle” environmental Life Cycle Assessment (LCA) was performed according to International LCA standards and the European standards on the environmental evaluation of buildings. These results were based on site-specific data and resulted from a consistent methodology, fully described in the paper for each life cycle stage: Cork oak tree growth, ICB production, and end-of-life processing-modeling of the carbon flows (*i.e.*, uptakes and emissions), including sensitivity analysis of this procedure; at the production stage—the modeling of energy processes and a sensitivity analysis of the allocation procedures; during building operation—the expected service life of ICB; an analysis concerning the need to consider the thermal diffusivity of ICB in the comparison of the performance of insulation materials. This paper presents the up-to-date “cradle-to-cradle” environmental performance of ICB for the environmental categories and life-cycle stages defined in European standards.

## 1. Introduction

The consumption of energy in the world today contributes to pollution, environmental degradation, and global greenhouse emissions. In the European Union (EU), the building sector is responsible for over 40% of overall energy consumption, making a significant contribution to CO_2_ emissions [1,2,3]. Thermal insulation materials have an important role, and their use is a logical first step to reduce the energy required to keep a good interior temperature, and therefore achieve energy efficiency [4] and reduce the energy, environmental, and economic impact of buildings [5]. An interdisciplinary research project was carried out to provide the environmental life cycle assessment of the production of the main thermal insulation materials of buildings, and their corresponding comparison [5].

**Figure 1:**
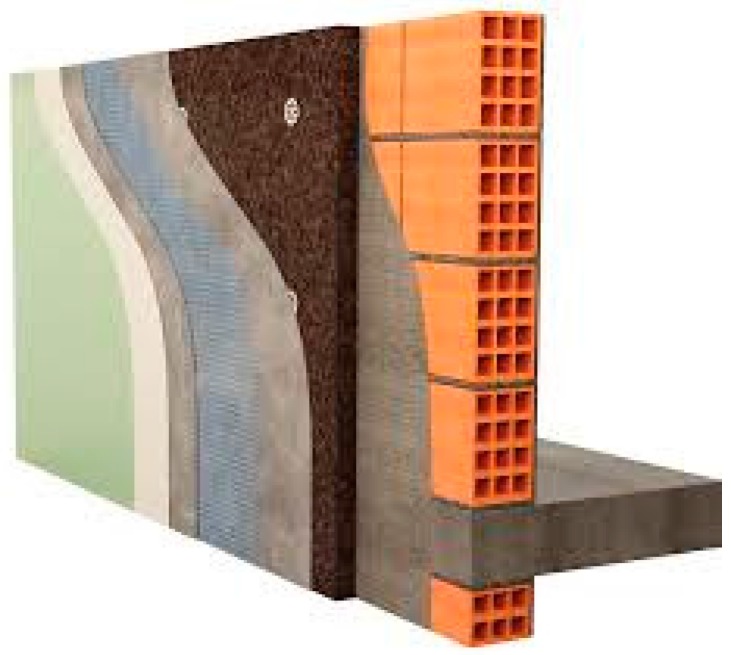
Insulation cork board (ICB) in an External Thermal Insulation Composite System (ETICS) [9].

**Figure 2:**
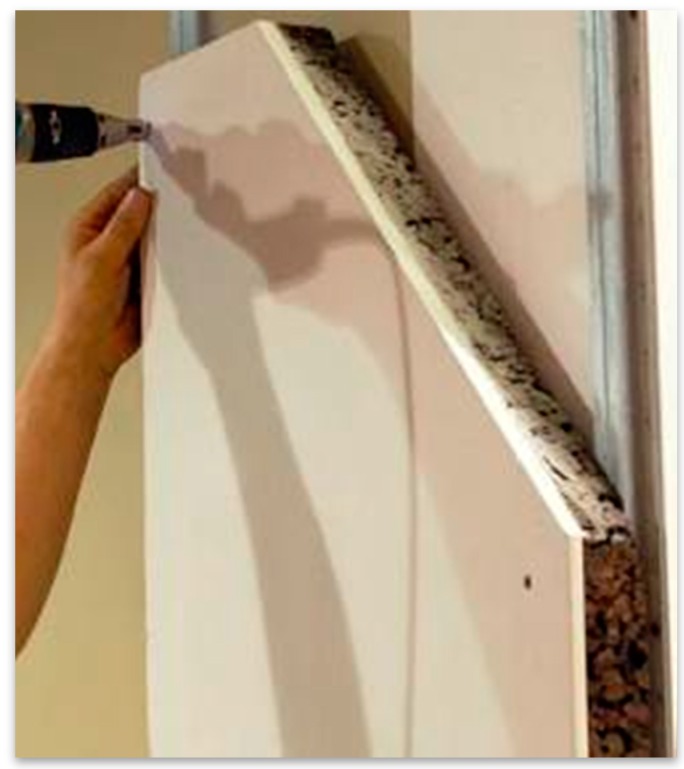
ICB glued to gypsum plasterboard in an Internal TICS (ITICS) [10].

Expanded cork agglomerate (Insulation Cork Board—ICB, or expanded cork agglomerate) is an insulation material that can be used in the envelope of buildings (Figure 1 and Figure 2). Portugal is the world’s largest producer and exporter of cork-based materials, including this cellular “organic natural” insulation material (ICB), but ICB is also produced in some other countries around the world [6]. Contrary to other organic materials, such as straw bale [7,8], ICB is used in construction all over the world, and not only in local or small-scale construction. The main technical characteristics of ICB studied (available thicknesses, density, declared thermal performance, and CE—“Conformité Européene” marking) are presented in Table 1.

There are some life cycle assessment (LCA)-based studies on cork materials that highlight the sustainability and environmental benefits of the cork oak tree forest, namely in fighting deforestation in arid zones and the capture of carbon for very long periods, as cork oaks are long-living trees (up to 200–250 years) [11,12,13]. However, the potential environmental advantages “from cradle-to-cradle” (C2C) of ICB are not yet reproduced in detailed LCA studies and need to be quantified using rigorous scientific methods in order to be unequivocally accepted at national and international levels. The aim of this paper is therefore to determine and evaluate the environmental impacts of the production, use, and end-of-life processing of ICB, based on real data obtained from a Portuguese manufacturer.

This paper comprises five sections, including this introduction. The Life Cycle Assessment (LCA) methodology used is described in detail in Section 2. The resulting figures for the production, use, and end-of-life of ICB are presented and analysed in the Section 3. Section 4 presents a discussion of the methodology used and results achieved in this paper, and the paper ends by drawing conclusions that summarize the main findings of the work.

## 2. Materials and Methods

The LCA approach used followed European standards developed under CEN/TC 350 [14,15], international standards on LCA [16,17], and some methodological procedures described in detail in this section.

### 2.1. Goals and Scope

The purpose of an LCA study and its field of application must be clearly defined. The goal of the current LCA study is to outline the environmental profile of ICB manufactured in Portugal using site-specific data, considering distinct methodology for biogenic CO_2_-accounting and disseminating the results, particularly in the scientific community involved in the development of insulation materials.

### 2.2. Declared Unit

The product studied is an insulation material mainly used for application in walls and roofs to promote both thermal and acoustic insulation. Thus, the common functional unit for ICB is the area of application of the insulation (m^2^). Nevertheless, the declared unit used in the LCA study was 1 m^3^ of ICB, and the environmental impacts are presented relative to this volume, since it is the reference that the producer uses to measure all manufacturing flows. Considering that ICB boards may have different thicknesses, the environmental impacts (*x*) of one square meter of this material with any *y* cm thickness will be:

*x* = (impacts relative to 1 m^3^) × *y*/100 (1)

The board density was considered to be 110 kg/m^3^ (Table 1).

### 2.3. System Boundaries

**Figure 3:**
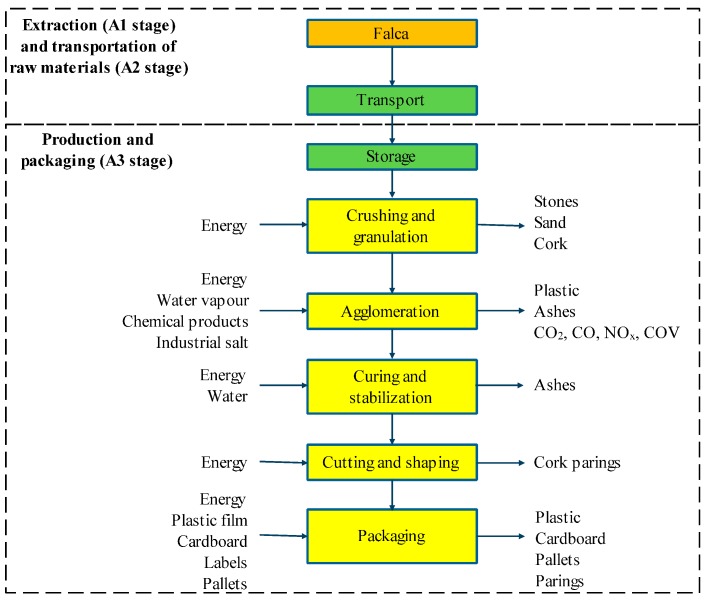
Main stages of ICB production and corresponding inputs and outputs.

The life cycle stages of construction materials and products are already standardized (Table 2) at the European level [14,15]. Therefore, the boundaries of an LCA study of a building material or assembly can be defined either from cradle-to-gate (including the extraction and processing of raw materials and production), from cradle-to-grave (including also the transport, distribution, and assembly, use, maintenance, and final disposal), or C2C, which further includes the reuse, recovery, and/or recycling potential (Table 2 and Figure 3) [18,19]. The system boundaries establish the unit processes to be included in the study. A C2C LCA approach is used in this paper, which means that the environmental impact analysis starts at the extraction of raw materials (A1 stage) and continues through the transportation and storage of raw materials (A2 stage), production, and packaging (A3 stage), based on site-specific data from ICB production. The use stage-operational energy use (B6) was not considered in this study, but is discussed in Section 3.3. The remaining stages of the life cycle after production (End-of-life stage (C1–C4) and Benefits and loads beyond the system boundary (D)) are characterised, assessed, and evaluated based on scenarios [20,21].

### 2.4. Cradle to Gate Life Cycle Inventory (LCI)

ICB are produced in an industrial plant located in the centre area of Portugal, 86 km from Lisbon (the Capital of Portugal). A questionnaire was prepared and sent to the producer to obtain the production data required for the LCI modelling. For a better understanding of the manufacturing (A3) processes, some visits to the ICB factory were made and several intermediate phases were identified. The LCI model is based on average production data from 2008 and 2010, since 2009 production was irregular, and therefore not considered in the model. Background data for modelling the production process was taken from the Ecoinvent database [22] (e.g., data for the extraction/production of raw and packaging materials, electricity, and transportation of raw materials, including the extraction of “falca”, the waste wood that results from periodic paring and pruning operations of the upper branches of cork oak trees [23], which modelling was made using “Raw cork, at forest road” process), but cultivation processes are out of the boundaries of the LCA study. In fact, the raw material used is a by-product or a waste from other product systems, and therefore the cultivation process is allocated to such systems (production of noble cork products). Moreover, in Portugal, wild oak tree forest is an autochthonous forest (protected by law) and the felling of cork oaks is not allowed except for essential thinning or to remove decrepit trees. No chemical products are used during the cultivation, which is a natural process, little influenced by humans [11]. All data used in the inventory phase were based on the questionnaire answered by the manufacturer. The LCA tool chosen to model the production process was SimaPro [24].

For reasons of transparency and traceability, and following the recommendations of European standards [14], the environmental impacts and potential benefits quantified in the A3 stage are subdivided in this paper into three independent information modules which set out the manufacturing process in more detail:

- A3.1—covering manufacturing and transportation to the factory of the packaging material that leaves the factory gate with the product;
- A3.2—covering the gate-to-gate manufacturing of the product being studied, and of ancillary materials, pre-products, and co-products, all internal transportation, and the disposal of final waste (except packaging waste) generated during production;
- A3.3—covering the production and disposal of raw materials or admixtures’ packaging, and of the wrapping material of the packaging products.

The production of packaging for raw materials or admixtures (and also of the material for wrapping the packaging products) was included in the A3.3 module rather than the A3.2 (or A3.1) modules because it was impossible to isolate each of the flows from the global packaging waste streams accounted for in the plant.

In an LCA study it is important to assess the quality of the site-specific data used. Ferrão [18] proposes a method to classify the quality of information used in an LCA study. This method (reproduced in Table 3) includes the most important indicators to evaluate the quality of data collected and was applied to the classification of the information used in the LCA study presented in this paper.

The life cycle inventory data used in this study was collected from the manufacture plant, so it was based on site specific data. From Table 3, it is possible to conclude that the quality of the information of this study (with a value of 1.6 in a 1 to 5 scale, where 1 = the best quality), can be considered a good and appropriate value for the global aim of this work.

In summary, the LCI was developed based on site specific data (for the production site processes). Nevertheless, during the modelling of the system process, modules from validated international databases (such as Ecoinvent [22]) were used for the background processes.

### 2.5. Choice of the Environmental Impact Assessment Method (EIAM) and Categories

According to the European standard that provides the core product category rules for all construction products and services, EN 15804:2012+A1 2015 [14], the impact assessment should involve seven categories:

- Global warming potential over a time span of 100 years (GWP);
- Ozone depletion (ODP);
- Acidification potential of soil and water (AP);
- Eutrophication potential (EP);
- Photochemical ozone creation potential (POCP);
- Depletion of abiotic resources (elements and fossil, separately, but the latter may be used and explained alone, if the values are known) (ADP).

The characterization factors were taken from CML 2001 (developed in The Netherlands by the Institute of Environmental Sciences (CML) of Leiden University). Therefore, this EIAM was chosen for the impact assessment of the product studied.

The results presented in this paper include two more environmental categories calculated based on a single issue method published by Ecoinvent and expanded by PRé Consultants [25]. The cumulative energy demand (CED) method expresses the depletion of energy resources and its calculation is based on the higher heating value [26]. It provides, in fact, the calculation of six environmental categories (non-renewable, fossil; non-renewable, nuclear; non-renewable, biomass; renewable, biomass; renewable, wind, solar, geothermal; renewable, water) which were grouped and presented in a simplified form in only two categories with the same unit (megajoule, MJ):

- Consumption of primary energy, renewable (PE-Re, or renewable energy resources depletion);
- Consumption of primary energy, non-renewable (PE-NRe, or non-renewable energy resources depletion).

## 3. Results

This section presents the “cradle-to-cradle” environmental LCA of ICB, including the cradle-to-gate environmental impacts, an analysis of the expected service life of ICB and of its environmental impacts at stages C1–C4 and D, depending on the building assembly where it is installed, and the consequences in the expected operational energy use of considering the thermal diffusivity of ICB.

### 3.1. Cradle-to-Gate (A1–A3) Environmental Impacts

**Figure 4:**
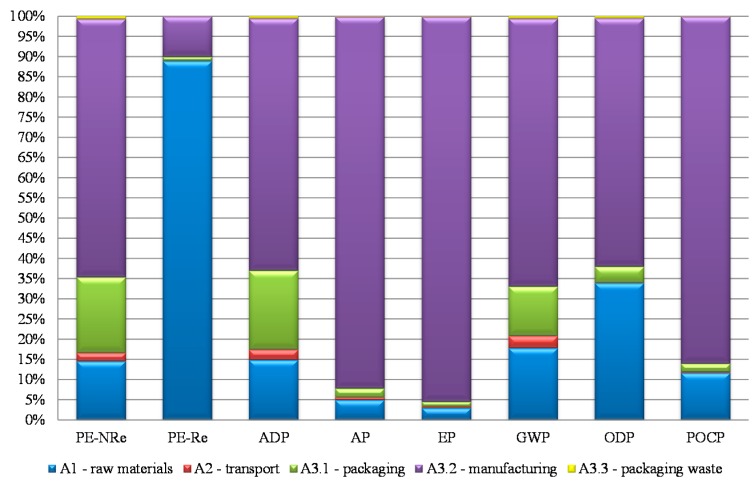
Relative contribution of each sub-stage of ICB production to environmental impacts [5]. PE-NRe: Consumption of non-renewable primary energy; PE-Re: Consumption of renewable primary energy; ADP: Depletion of abiotic resources; AP: Acidification potential of soil and water; EP: Eutrophication potential; GWP: Global warming potential over a 100-year span; ODP: Ozone depletion; POCP: Photochemical ozone creation potential.

Figure 4 shows the relative percentage contribution of each sub-stage (A1–A3) for the cradle-to-gate environmental impacts of ICB and reflects the fact that only one raw (and natural) material is used in ICB production-“falca”. Therefore, the A1 sub-stage contribution is significant only for PE-Re (88.9%) and for ODP (33.9%), the former being mainly related to forests and forest roads, conservation, and maintenance operations to allow raw material extraction. However, the contribution of manufacturing (A3.2) is significant (more than 65%) in many categories, such as AP, EP, GWP, and POCP. Looking in more detail at the individual contributors to A3.2 sub-stage impacts for EP, it is found that the most important contribution to this category (about 40%) corresponds to the direct air emissions from the boiler during heating of water for the expansion process. Electricity consumption contributes around 10% to EP, while the disposal of the wood ash residue from the boiler for use on agricultural land is responsible for 48.2% of the impacts in this impact category. Concerning the individual contributors to A3.2 sub-stage impacts for GWP, only electricity consumption has a significant impact (95.8%) because the CO and CO_2_ emissions from the boiler and from the autoclave are biogenic and thus not considered in this impact category by the EIAM used (CML) [5].

Cradle to gate LCA results of the production of one cubic metre of ICB are presented in Table 4.

### 3.2. B4—ICB Replacement (Depending on the Building Assembly)

The minimum durability (or expected service life) of ICB for insulation has recently been estimated as 50 years without decay in thermal performance for normal application in buildings. In fact, the National Civil Engineering Laboratory (LNEC) tested samples of ICB with this age and in good condition removed from a building being refurbished and found a thermal conductivity of 0.0392 W/m·K (lower than the current one, Table 1) [27]. Therefore, no replacement of ICB has to be planned during the 50-year service life of a building, nor its maintenance, when applied as an insulation (e.g., in an external or internal insulation system or inside a cavity wall). Nevertheless, stochastic data (if available) from service life prediction (SLP) studies should be used in C2C LCA of ICB external cladding because of its unforeseen durability and complex pathologic process [28].

### 3.3. B6—Use Stage—Operational Energy Use

The use of ICB in different assemblies of the envelope of buildings can reduce the operational energy use due to its low thermal conductivity, but also because of its low thermal diffusivity. However, while the thermal conductivity is always considered in building design or by thermal regulations, the consideration of the thermal diffusivity of insulation materials applied in an external building assembly requires the analytical calculation of the thermal delay of multi-layered systems. This requirement prevents the consideration of the thermal diffusivity, and of the corresponding thermal delay of the external wall or roof, on a regular basis.

If the thermal diffusivity is also considered in the comparison of the environmental, economic, and energy performance of insulation materials used in a buildings envelope, the use of ICB would lead to a thermal delay of the assembly 1.5 times higher than that of other concurring materials with the same thickness (but with higher thermal diffusivity) [29]. However, the quantification of this advantage of ICB in the three dimensions of performance referred would only be possible by: Considering a specific building and considering different insulation materials applied in an external assembly (taking into account the resulting thermal decays); providing a dynamic thermal simulation of the performance of the building during a whole year to calculate the corresponding heating and cooling needs; applying a method to provide an assessment of the life cycle performance from cradle-to-cradle of these assemblies in these dimensions, such as 3E-C2C [30,31].

### 3.4. C1–C4—End-of-Life Stage, and D—Benefits and Loads Beyond the System Boundary

The environmental impacts and loads of ICB at stages C1–C4 and D depend on the building assembly of the envelope where it is installed. In fact, as referred to before, 50-year-old samples of ICB were tested and presented a thermal conductivity similar to a new board. These samples were in good condition, except for the damage caused by demolition, and were cleaned of adhesive or stone wastes and fully recycled by granulation and placed in the market as regranulate of ICB [27]. Therefore, it can be said that ICB applied in cavity walls, for example, can be completely recycled and reused as granulate at end-of-life. However, when ICB is applied as a component of an ETICS or ITICS system, it cannot be physically separated from the remaining ones, and it has to be considered that they are mixed after demolition, *i.e.*, considered as undifferentiated construction and demolition waste (CDW; waste code 17 09 04—mixed construction and demolition wastes [32]) and sent to landfill.

Environmental impacts were calculated in both cases for stages C and D (Table 2), considering:

- The transportation of the discarded product as part of the waste processing (C2, e.g., to a recycling site) and transportation of waste (C2, e.g., to final disposal);
- The waste processing (C3, e.g., collection of waste fractions from the deconstruction and waste processing of material flows intended for reuse, recycling, and energy recovery);
- The waste disposal, including physical pre-treatment and management of the disposal site (C4);
- The benefits and loads beyond the system boundary (3R potential of CDW and of other waste flows).

The environmental impacts of demolition (sub-stage C1—deconstruction, including dismantling or demolition, of the product from the building, including initial on-site sorting of the materials) were not considered since they are similar for all the alternatives under assessment. The environmental impacts of transporting and disposing of the CDW were based on Portuguese case studies which used data from waste operators [33]. Waste treatment processes from the Ecoinvent database were used to model the referred to disposal of each waste stream generated.

Table 5 shows the environmental benefits of ICB recycling at end-of-life, which can result in savings of 4% of the consumption of non-renewable primary energy in the production of these boards and represent 5% of the cradle-to-gate GWP, due to avoiding “falca” extraction (but considering the transport from the construction site to the plant). However, when ICB is a part of an internal or external insulating system, its transportation and landfilling have to be added to the cradle-to-gate environmental impacts, and can represent between 1% (in AP) and 71% (in EP) of these impacts in the most important categories.

## 4. Discussion

This section presents a discussion of the methodology used and of the results achieved in this paper, namely for the product and end-of-life stages.

### 4.1. Cradle-to-Gate (A1–A3) Environmental Impacts

To the best of the authors’ knowledge, there is yet no complete LCA study available worldwide concerning ICB apart from the one presented in detail in this paper. An Environmental Product Declaration (EPD) was already published in the Portuguese EPD Programme in 2015, but it is a cradle-to-gate study [23]. The LCA results in this paper are however original in international terms, in that no LCA data set was identified for very similar (nor similar) products on a cradle-to-cradle approach. In this case, the extraction of the only raw material of ICB-“falca”-was modelled using a process from Ecoinvent (as referred) that is based on data from Germany and Portugal extrapolated for Europe. This process is used in Ecoinvent to model the production of a “cork slab” that is “used as underlay for floating floorings or as insulation material”.

Data for the production of this cork slab was collected from a “major producer in Portugal”, but it must be referred that cork “underlays” and ICB are produced in different plants using different raw materials. While the former use cork waste from cork wine stopper production agglomerated with resins, the latter use “falca” (the waste wood that results from periodical paring and pruning operations of cork oak trees [23]) and no additional admixtures or artificial resins. Therefore, the “cork slab” process of Ecoinvent corresponds to a mixture of production processes from different plants, their disaggregation being impossible to a practitioner without having access to the complete information concerning the data collection.

“Raw material extraction and processing, and processing of secondary material input” (A1) makes a significant contribution to the cradle-to-gate environmental impacts of several insulation materials [5], but that is not the case of ICB, which is based on natural raw materials and the production of which promotes recycling of agricultural waste (Table 4). The LCA results of the ICB “product stage” also show that some life cycle stages, such as transportation of raw materials (A2), packaging and packaging waste (A3.1 and A3.3, respectively), may not be discarded in a cradle-to-gate study of a construction material because they can make a significant contribution to some environmental categories (Figure 4).

In this case, with the exception of PE-Re (which is more relevant in the extraction stage because the raw material is biomass, included in the renewable energy resources), the manufacturing stage has the highest impact in all categories, especially in acidification and eutrophication potentials (Figure 4).

### 4.2. A1–A3—Carbon Dioxide Flows (Uptakes and Emissions)

**Figure 5:**
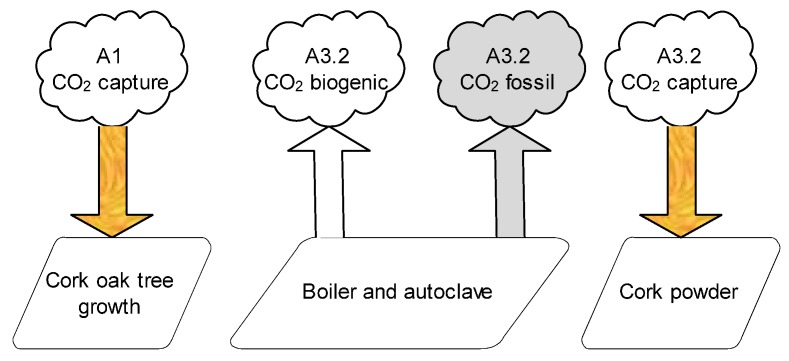
CML only considers CO_2_ fossil emissions (in grey) and does not consider CO_2_ capture during tree growth or biogenic CO_2_ emissions.

The GWP figures for the “product stage” (A1–A3) of ICB presented in Table 4 did not consider the CO and CO_2_ emissions from the boiler and from the autoclave. These emissions are biogenic and are presently not considered by the most used EIAM (including the most recent version of CML). The same occurs with the CO_2_ captured during cork oak tree growth, namely by “falca” used in ICB production and cork powder bought externally for the boiler (Figure 5).

However, ICB raw material is renewable and able to capture CO_2_ from the atmosphere, and a significant quantity of biogenic CO_2_ is released to the atmosphere during ICB production, even though these flows are not considered in the most recent LCA studies. Therefore, Table 6 shows a sensitivity analysis of the consequences of this methodological choice, already supported by BRE Product Category Rules (PCR) for construction Products EPD [34], as well as by CEN/TC 350 standards EN 16449:2014, EN16485:2014 and Fpr CEN/TR 16970:2015 [35,36,37]. Some of these documents also include detailed formulas for the calculation of biogenic carbon captured/released by biomass material [34,35,36]. It was found that cradle-to-gate (A1–A3) GWP results are very dependent on the methodological procedure chosen. In this specific situation, the calculation method for embodied carbon used, using a process from the Ecoinvent database [22] (1.7 kg·CO_2_ eq/kg of processed cork after A1 processes, and a higher value for raw cork at extraction), was found to be less conservative than the guidelines for calculation included in the referred standards (which consider around 1.74 kg·CO_2_ eq/kg of raw cork) [34,35,36].

Regardless, considering CO_2_ capture for “falca” leads to a significant negative balance of −435 kg·CO_2_ eq per cubic meter of ICB, while the figure achieved using CML was positive (40.2 kg·CO_2_ eq). Considering biogenic CO_2_ emissions is not sufficient to offset CO_2_ uptake, but the result for GWP during manufacturing (A3.2) increases almost seven times (even considering CO_2_ uptake by the cork powder bought externally).

### 4.3. A3 (Manufacturing)—Allocation Procedure

The requirements for the allocation procedure to be considered in LCA studies are included in international and European standards [14,16,17]. These requirements were taken into account when modelling ICB production to allow the artificial division of the input and output flows (and relevant environmental impacts) of the operation of the plant by the different products manufactured in order to assign a proportion to the product system under study. A summarized description of the allocation procedure followed in the LCA study is presented in this section, including a comparison of the consequences of the choice of a physical (e.g., volume or mass) or economic allocation.

The allocation procedure is most critical for products that are co-produced with other goods and/or for which manufacturing results in production waste that is recycled inside the plant and sold as a co-product. The production of ICB includes the latter situation because it co-produces regranulate from the milling of production waste. Three allocation alternatives were considered for this manufacturing process: Volume, mass, and economic allocation (Table 7). The first option is the most obvious and direct, because all production flows are measured by the company based on the final production volume of each product (boards and regranulate). However, allocation based on the final volume does not express the physical relationship between the products during the production process (the density of the boards is 110 kg/m^3^ and the bulk density of regranulate is 70 kg/m^3^). The option was therefore to apply mass allocation (using the final production volume and corresponding density or bulk density) between these two products in order to correctly express the physical relation between them during manufacturing [5].

Allocation can also be economic, especially when the difference in revenue from the co-products is not low, which can be estimated at 9% or more (1% or less is considered very low and more than 25% is regarded as high, according to European standards (CEN, 2012)). In this case, the difference in revenue between ICB boards and regranulate is around 27%, which is high. Taking into account the proceeds from these revenues [38], it was found that economic allocation can increase the share of ICB boards by 4% (Table 7). However, this alternative was not selected because it leads to final results that do not respect the underlying physical relationships between the products. Moreover, LCA results achieved using economic allocation do not express the authentic environmental impacts related to the production of each co-product. Furthermore, these results cannot be compared with available LCA results for the same products (in LCA databases or EPD) because the latter are usually achieved using allocation based on physical relations [5].

### 4.4. A3.2—Energy Processes

Processes included in the Ecoinvent database [22] for each energy carrier appropriately represent the reality of Western countries, including Portugal, namely the interdependent network between countries that characterizes the international trade in electricity [39]. Therefore, these were used as a basis to model the energy supply of the production processes studied, while the corresponding quantification was carried out using site-specific data. Based on a specific composition of these energy carriers, the Ecoinvent database also includes processes that correspond to the national electricity supply for industrial (Electricity, medium voltage, at grid/PT U) consumers, based on the energetic mix of 2004. However, to accurately estimate the environmental impacts in terms of energy consumption for the production of ICB, these processes were updated using the latest information available concerning the Portuguese electricity mix (data from 2011) [40]. The processes themselves were not actually modified; rather, their share in the national electricity mix. The use of the national electricity mix that expresses the present reality is even more important when the manufacturing (A3) is energy intensive like in ICB production, and, indeed, most of the environmental impacts of the life cycle of the product come from this stage [5].

### 4.5. C2–C4 and D—Carbon Dioxide Flows (Uptakes and Emissions)

The GWP figures for the “End-of-life stage” (C2–C4 and D) of ICB presented in Table 5 did not consider the CO_2_ captured during cork oak tree growth, namely by the “falca” that is used in ICB production. However, if this flow is considered, the result will not be the most desired for the ICB from a cavity wall: Instead of saving GWP, the ICB recycling at end-of-life will result in a significant impact in this category (1.86 × 10^2^ kg CO_2_ eq) because of the reuse of demolition waste replacing a raw material that captures CO_2_. The consideration of biogenic CO_2_ emissions in landfilling increases around 30% the GWP result for ICB in ITICS or ETICS system.

## 5. Conclusions

The cradle-to-gate LCA results per life cycle stage and environmental category of Insulation Cork Boards (ICB) have been presented and analyzed, along with the identification of the processes that contribute most to each category. A sensitivity analysis was performed to evaluate the consequences in LCA results of: Physical and economic allocation; carbon flows (*i.e.*, uptakes and emissions); the expected service life and thermal diffusivity of ICB. The latest information available on the Portuguese electricity mix was considered to accurately estimate the environmental impacts of the company arising from the consumption of energy for the production of ICB.

It was concluded that the methodology used in the biogenic carbon account should not be determinant in a C2C approach, since the final balance should be null when closing the cycle. The manufacturing stage showed the most relevant impacts for all categories but PE-Re, mainly because of the high impacts of electric energy production.

Considering only the A1–A3 stages of the life cycle, ICB has a low contribution to several impact categories when compared to its direct competitors (expanded and extruded polystyrene, mineral wool, and polyurethane), namely in PE-NRe (which means that the production of ICB requires low consumption of fossil fuels), ADP, and GWP, while in PE-Re and water consumption ICB has a higher impact [5,41]. Depending on the criteria of the specifier, and on the sources of electric energy, ICB may be a sustainable choice for building insulation from an environmental point of view.

The LCA results presented here are considered scientifically sound since they were achieved through a consistent methodology (described in detail in the paper), which also takes into account the most recent European standards, and subjected to a full set of sensitivity analysis processes. These results are also innovative and up-to-date on an insulation product for use in buildings, in particular because they include for the first time the “cradle-to-cradle” environmental performance of ICB.

## Figures and Tables

**Table 1:** Main technical characteristics of ICB studied in this paper.

Product	Available Thicknesses (mm)	Density (kg/m^3^)	Declared Thermal Performance-λ (W/(m·K))	CE Marking (Standard)
ICB	40–150	110	0.04	Yes (EN 13170:2012+A1:2015—Thermal insulation products for buildings—Factory made products of expanded cork (ICB)—Specification)

**Table 2:** Detailed life cycle stages of building materials classification based on European standards.

LCA Boundaries			Life Cycle Stages/LCA Information Modules	Life Cycle Stage Designation and Description
Cradle to cradle	Cradle to grave	Cradle to gate	Product stage (A1–A3)	A1—raw material extraction and processing, processing of secondary material input; A2—transport to the manufacturer; A3—manufacturing
		Gate to grave	Construction process stage (A4–A5)	A4—transport to the building site A5—installation in the building
			Use stage—information modules related to the building fabric (B1–B5)	B1—use or application of the installed product; B2—maintenance; B3—repair; B4—replacement; B5—refurbishment
			Use stage—information modules related to the operation of the building (B6–B7)	B6—operational energy use B7—operational water use
			End-of-life stage (C1–C4)	C1—de-construction, demolition; C2—transport to waste processing; C3—waste processing for reuse, recovery and/or recycling (3R); C4—disposal
			Benefits and loads beyond the system boundary (D)	D—reuse, recovery and/or recycling (3R) potential

**Table 3:** Quality of the information used in an LCA study (adapted from [18]) and in the Life Cycle Inventory (LCI) of the ICB studied in this paper (adapted from [5]).

Scale	Confidence	Integrity	Temporal Correlation	Geographic Correlation	Technological Correlation
1	Verified ^a^ data and based on measurements ^b^	Data representing sufficient ^c^ number of companies over a period that enables the elimination of fluctuations	Maximum difference of 3 years from the year under study	Data from the region under study	Data from the company under study
2	Partially verified data and based on hypothesis ^d^, or not verified but based on measurements	Data representing a small number of companies, but for appropriate periods	Less than 6 years difference	Average data from a region larger than that under study, but including it	Data from the same processes/materials but from other companies
3	Unverified data and partially based on hypothesis	Data representing a suitable number of companies, but for short periods	Maximum difference of 10 years	Data from a region with similar production conditions	Data from the same processes/materials but from a different technology
4	Verified or qualified estimations (produced by experts)	Representative data, but from a small number of companies and from short periods, or incomplete data from a suitable number of companies and period durations	Difference less than 15 years	Data from a region with production conditions with some similarities	Data from similar processes/materials but analogous technology
5	Neither verified nor qualified data estimations	Unknown representativeness, or incomplete data from a small number of companies and/or short periods	Unknown age of data or difference more than 15 years	Data from an unknown region, or from a region with very different production conditions	Data from similar processes/materials but different technology
Company that produces ICB (1.6)	2—Unverified (but including a visit to the production line), but based on measurements	2—One company and a two-year period; market share (%)—most important company in the national market	2—2008 and 2010	1	1

^a^ Data can be verified by comparison with original documents, by repeating the calculations, by comparison with other sources, by material or energy balances, *etc.*; ^b^ Experimental measurement techniques must be described in the report; ^c^ In order to be statistically representative, data need not be complete. However, the chosen sample must be randomly chosen and be of an appropriate size to be reproducible and truly reflect the characteristics of the whole population; ^d^ The considered hypothesis in the collection of inventory data must also be specified in the report.

**Table 4:** LCA results for each sub-stage of the “product stage” (A1–A3) of one cubic metre of ICB (with a density of 110 kg/m^3^).

Category Indicator	Unit	Life Cycle Stages (Total per m^3^)					
		A1–A3	A1	A2	A3.1	A3.2	A3.3
PE-NRe	MJ	8.21 × 10^2^	1.19 × 10^2^	1.73 × 10^1^	1.54 × 10^2^	5.25 × 10^2^	5.56
PE-Re	MJ	7.68 × 10^3^	6.83 × 10^3^	2.31 × 10^−2^	7.53 × 10^1^	7.74 × 10^2^	1.18
ADP	kg Sb eq	3.31 × 10^−1^	4.91 × 10^−2^	8.47 × 10^−3^	6.47 × 10^−2^	2.07 × 10^−1^	1.76 × 10^−3^
AP	kg SO_2_ eq	9.05 × 10^−1^	4.47 × 10^−2^	5.80 × 10^−3^	1.99 × 10^−2^	8.34 × 10^−1^	1.07 × 10^−3^
EP	kg PO_4_^3-^ eq	4.03 × 10^−1^	1.19 × 10^−2^	1.33 × 10^−3^	4.87 × 10^−3^	3.84 × 10^−1^	4.65 × 10^−4^
GWP	kg CO_2_ eq	4.02 × 10^1^	7.15	1.23	4.92	2.67 × 10^1^	2.33 × 10^−1^
ODP	kg CFC-11 eq	2.78 × 10^−6^	9.43 × 10^−7^	2.33 × 10^−9^	1.12 × 10^−7^	1.71 × 10^−6^	1.37 × 10^−8^
POCP	kg C_2_H_4_	6.38 × 10^−2^	7.43 × 10^−3^	1.36 × 10^−4^	1.33 × 10^−3^	5.49 × 10^−2^	4.71 × 10^−5^

**Table 5:** LCA results for the End-of-life stage (C2–C4) and for Benefits and loads beyond the system boundary (D) of one cubic metre of ICB (with a density of 110 kg/m^3^), and comparison with A1–A3 (Table 4).

Category Indicator	Unit	C2–C4; D (Total per m^3^)	
		ICB in Cavity Walls (% of A1–A3)	ICB in ITICS or ETICS System (% of A1–A3)
PE-NRe	MJ	−3.24 × 10 (−4%)	4.15 × 10 (5%)
PE−Re	MJ	−2.05 × 10^−3^ (−27%)	5.77 × 10^−1^ (0%)
ADP	kg Sb eq	−1.31 × 10^−2^ (−4%)	1.79 × 10^−2^ (5%)
AP	kg SO_2_ eq	−1.23 × 10^−2^ (−1%)	9.95 × 10^−3^ (1%)
EP	kg PO_4_^3−^ eq	−3.30 × 10^−3^ (−1%)	2.88 × 10^−1^ (71%)
GWP	kg CO_2_ eq	−1.91 (−5%)	6.88 (17%)
ODP	kg CFC-11 eq	−2.83 × 10^−7^ (−10%)	3.41 × 10^−7^ (12%)
POCP	kg C_2_H_4_	−2.21 × 10^−3^ (−3%)	1.87 × 10^−3^ (3%)

**Table 6:** GWP for each sub-stage of the “product stage” (A1–A3) of one cubic metre of ICB (with a density of 110 kg/m^3^) —Comparison between CML and the consideration of CO_2_ capture and biogenic CO_2_ emissions. EIAM: Environmental Impact Assessment Method.

Method	Category Indicator	Unit	Life Cycle Stages (Total per m^3^)							
			A1–A3	A1	A2	A3.1	A3.2—Fossil CO_2_ Emissions	A3.2—Biogenic CO_2_ Emissions	A3.2—CO_2_ Capture	A3.3
EIAM CML (Table 4)	GWP	kg CO_2_ eq	40.2	7.15	1.23	4.92	26.7	–	–	0.233
Consideration of CO_2_ capture and biogenic CO_2_ emissions			−435	−620	1.23	−1.12	(Total of 185)			0.170
							26.7	222	−63.6	

**Table 7:** Manufacturing share of ICB boards and ICB regranulate depending on the allocation procedure [5].

Allocation Procedure	Manufacturing Share (%)	
	ICB Boards	ICB Regranulate
Volume	75	25
Mass	83	17
Economic	87	13

## Abbreviations

The following abbreviations are used in this manuscript:

AP	Acidification Potential
ADP	Abiotic Depletion Potential
C2C	Cradle-to-cradle
CDW	Construction and Demolition Waste
CED	Cumulative Energy Demand
EIAM	Environmental Impact Assessment Method
EP	Eutrophication Potential
EPD	Environmental Product Declaration
ETICS	External Thermal Insulation Composite System
EU	European Union
GWP	Global Warming Potential over a time span of 100 years
ICB	Insulation Cork Board
ITICS	Internal Thermal Insulation Composite System
LCA	Life Cycle Assessment
LNEC	National Civil Engineering Laboratory
ODP	Ozone (stratospheric) Depletion Potential
PE-Re	Consumption of primary energy, renewable (or renewable energy resources consumption)
PE-NRe	Consumption of primary energy, non-renewable (or non-renewable energy resources depletion)
POCP	Photochemical Ozone Creation Potential
